# American Sign Language Words Recognition of Skeletal Videos Using Processed Video Driven Multi-Stacked Deep LSTM

**DOI:** 10.3390/s22041406

**Published:** 2022-02-11

**Authors:** Sunusi Bala Abdullahi, Kosin Chamnongthai

**Affiliations:** 1 Department of Computer Engineering, Faculty of Engineering, King Mongkut’s University of Technology Thonburi, Bangkok 10140, Thailand; sbabdullahi@ieee.org; 2 Zonal Criminal Investigation Department, The Nigeria Police, Louis Edet House Force Headquarters, Shehu Shagari Way, Abuja 900221, Nigeria; 3 Department of Electronic and Telecommunication Engineering, King Mongkut’s University of Technology Thonburi, Bangkok 10140, Thailand

**Keywords:** American sign language words, bidirectional long short-term memory, computer vision, deep learning, dynamic hand gestures, leap motion controller sensor, sign language recognition, ubiquitous system, video processing

## Abstract

Complex hand gesture interactions among dynamic sign words may lead to misclassification, which affects the recognition accuracy of the ubiquitous sign language recognition system. This paper proposes to augment the feature vector of dynamic sign words with knowledge of hand dynamics as a proxy and classify dynamic sign words using motion patterns based on the extracted feature vector. In this method, some double-hand dynamic sign words have ambiguous or similar features across a hand motion trajectory, which leads to classification errors. Thus, the similar/ambiguous hand motion trajectory is determined based on the approximation of a probability density function over a time frame. Then, the extracted features are enhanced by transformation using maximal information correlation. These enhanced features of 3D skeletal videos captured by a leap motion controller are fed as a state transition pattern to a classifier for sign word classification. To evaluate the performance of the proposed method, an experiment is performed with 10 participants on 40 double hands dynamic ASL words, which reveals 97.98% accuracy. The method is further developed on challenging ASL, SHREC, and LMDHG data sets and outperforms conventional methods by 1.47%, 1.56%, and 0.37%, respectively.

## 1. Introduction

Among sign languages, which are normally used in deaf communication, American sign language (ASL) is one of the standard [1,2,3] and popularly used sign language across the world. ASL words are performed using single and double hands in the deaf communication, and majority of ASL words are performed using double hands, which are dominant and non-dominant hands [4,5]. Several single-handed words have now added a second hand in an identical or reciprocal rotation, to increase redundancy. Such redundancy is a significant parameter in sign to discriminate similarity and to predict other parameters [6]. These double-hand sign words share some similar features, which usually occur at the beginning and ending of sign trajectory, which leads to misunderstandings. Most double hand sign words are dynamic words. Classification of dynamic sign words using single and double hands is the basic function for automatic sign language recognition applications; especially, the recognition of similar double hand sign words is an important and useful research problem in terms of accuracy.

It is observed from available existing works that sign words recognition has been performed using single or double hands and can be classified into deep learning and feature-based systems, as illustrated in Table 1. The deep learning approach recently emerged and can deal excellently with problems related to big data [7,8,9,10,11]. However, some problems may not be convenient for collecting a huge number of samples such as big data; thus, a feature-based system is introduced, in which users consider features in advance, and it performs well in some cases. The feature based-system for sign word recognition can be categorized into two groups: static and dynamic sign word groups. Research works in static sign words recognition [12,13] can recognize sign words to a high degree of accuracy but may not work well with dynamic sign words since the majority of dynamic sign words express their meaning in motion. Research works in dynamic sign word recognition are grouped into single- and double-hand dynamic sign words. Works in single-hand [5,14,15,16,17] dynamic sign words are limited due to the fact most of the sign letters or alphabets are less complex. Double-hand sign words are commonly used in daily communications, but complex hand motion interactions are major challenge. Recognition of double-hand both static and dynamic Indian gestures is proposed in [18]. The authors developed a system of utilizing feature points engineered from a minimum Eigen value algorithm to recognize double-hand, which was later converted using COM server in MATLAB as both text and speech. The method is limited to only statistical features. Demrcioglu et al. [19] designed a double-hand sign words recognition system from hand shapes, using three machine learning classifiers, among which the heuristics classifier outperformed others with good recognition accuracy, but the method suffers from insufficient features representation since the majority of double-hand actions are characterized by hand shape, motion and orientation. The work in Deriche [20] proposed the CyberGlove with the SVM model for double-hand dynamic word recognition. This method achieved good recognition; however, Cyberglove for sign words recognition is expensive, intrusive, and has imprecise calibrations. Haque et al. [21] designed a Two-Handed Bangla Sign Language Recognition system that recognizes 26 sign gestures, from a three-structured flow. Their method extracts images using Principal Component Analysis (PCA) and K-Nearest Neighbors (k-NN), which are used as classification algorithms. This method achieved a success rate of 77.8846% by testing 104 images. The major disadvantage of this method is low accuracy because of a complex camera background, as well as a limited number of considered features, while Raghuveera et al. [22] proposed ensemble learning using SVM from SURF, HOG and LBP hand features, to control complex camera background. This method achieved low accuracy because of non-scalable features, similarity, and complex segmentation. Karaci et al. [23] presented ASL letters using LR, k-NN, RF, DNN, and ANN classifiers for double-hand sign language recognition. The overall result of these experiments using cascade voting achieved an accuracy of 98.97%. The system can be useful for finger-spelling/letter signs only. The ASL recognizer system cannot be considered as a complete SLR system because we have to include dynamic sign words. Meanwhile, ref. [24] developed a system for Turkish dynamic sign word recognition based on multi-layer kernel-based extreme learning machine (ML-ELM) algorithm. The proposed method was capable of successfully recognizing sign words in the dictionary with an accuracy of 98%. The primary disadvantage of using ML-KELM is the problem of obtaining a least square optimal solution of ELM. A double-hand SLR application system using LMC with a Windows platform is proposed as an expert system in [25]. Hisham and Hamouda [26] built double and single-hand sign words recognition inside Latte Panda with an Ada-Boosting strategy. The method achieved good recognition but cannot learn sequential data and may fail with complex sign words. Researchers identified the potential of using recurrent neural network and its variants to effectively learn long-term dependencies for sign language recognition [27,28,29]. However, single LSTM has weak learning ability [30,31], and it falls easily into over-fitting, in contrast to multi-LSTM network [29]. A similarity problem of double-hand dynamic sign words is addressed by Avola et al. [32] using multi-stacked LSTM learning; they utilized 3D hand internal angles, position displacements of the palm, and the fingertips Equation (5) to recognize dynamic ASL words [32]. The work in [32] is good at recognizing some dynamic ASL words, but the major disadvantage of achieving large abstraction (deeper network) via the multi-stacked LSTM method is that learning ability is marginal when the sample feature is increased, and, consequently, the recognition rate does not significantly improve. However, ref. [32] considers a limited number of ASL dynamic words, and their handcrafted features are not sufficient to recognize most available dynamic ASL words, especially sign words from similar class. Thus, these models/algorithms are insensitive to human hand dynamics and cannot use various classes of features, which leads to bad extensibility. These two problems may lead to misclassification of double-hand dynamic ASL words. We observed that the existing methods failed to utilize about 7% of the first few video frames during segmentation. These discarded frames contain a hand pause feature, which is not properly processed by the existing recognition methods.

For this reason, we propose to utilize the 3D extended kalman filter (EKF) covariance matrix feature representation of double-hand motion trajectories and to add a hand pause feature, as our feature vector for double-hand dynamic sign words recognition. Skeletal videos from LMC are affected by noise, and we deploy a robust weighted least square (WLS) algorithm where each sequence is allocated with effective weights to obtain the best confidence score with the fewest residuals. The corrected video sequences are fed into the EKF to track 3D double-hand motion trajectories across video frames through estimating anonymous features by approximating a probability density function over the entire video sequence. Basic hand features (hand shape, orientation, position and motion) are automatically extracted from skeletal hand-joint videos using bi-directional recurrent neural network (BiRNN). The extracted features are transforms using maximal information correlation (MIC) and rows concatenation for best feature representation. Finally, the selected features are computed using video frames correction to control initial frame coordinates and positions. To this end, we design a consolidated feature vector to achieve flexible and effective learning of double-hand complex gesture recognition. Moreover, none of the existing literature has tried to use the performance of networks to optimize loss function. This paper intended to bridge this gap. In addition to the mentioned research focus, dynamic hand gesture recording and recognition was applied in various consumer applications [33,34,35]. We made the following contributions:

(a) Acquisition and processing of skeletal video images acquired by means of a portable leap motion controller (LMC) sensor.
(b) The development of an EKF-tracking to address hand motion tracking errors and uncertainties across each frame in obtaining hand motion trajectories.
(c) The development of an innovative algorithm based on WLS to control noise across video frames.
(d) The design of a BiRNN network that is able to extract the proposed features from raw skeletal video frames.
(e) The development of an MIC scheme to select the most significant features from raw video images. These are used as input to the multi-stacked deep BiLSTM recognition network to discriminate among similar double-hand dynamic ASL words.
(f) Intensive evaluation using Jaccard, Mathew correlation and Fowlkes–Mallows indices is carried out to analyze the reliability of recognition results. These indices estimate the confusion matrix via known parameters for assessing the probability that the performance would be achieved by chance, due to the assumption of randomness of the k-fold and LOSO cross-validation protocol.
(f) Investigation of the best recognition network by comparing the performance of Adam, AdaGrad and Stochastic gradient descent on loss function, for ubiquitous applications.

The remainder of our article is structured as follows: Section 1 provides relevant works; Section 2 provides basic feature definitions, skeletal video preprocessing, WLS, hand tracking using EKF, MIC, features-scaling, skeletal-video-frames correction, ASL words recognition from skeletal video feature, BiRNN features extraction, LSTM, model parameters, evaluation metrics, experiments, and data set design; Section 3 provides results and details of a performance comparison with baseline methods; Section 4 discusses the implemented approach; and Section 5 concludes the entire work.

**Table 1:** Table of Related works.

Algorithm Name	Methodology	Results (%)	Limitations
1. DEEP LEARNING-BASED SYSTEM			
Konstantinidis et al. [8]	Meta-learner + stacked LSTMs	99.84 and 69.33	Computational complexity
Ye et al. [10]	3DRCNN + FC-RNN	69.2 for 27 ASL words	annotation and labeling is required
Parelli et al. [36]	Attention-based CNN	94.56 and 91.38	large-scale data set required
Asl-3dcnn [11]	cascaded 3-D CNN	96.0, 97.1, 96.4	spatial transformation
B3D ResNet [7]	3D-ResConVNet + BLSTM	89.8 and 86.9	Misclassification + large data require
Rastgoo et al. [9]	SSD + 2DCNN + 3DCNN + LSTM	99.80 and 91.12	complex background + large data
2. FEATURE-BASED SYSTEM			
2.1 Static sign words			
Mohandes et al. [12]	MLP + Naïve Bayes	98 and 99	Not very useful for daily interact
Nguyen and Do [13]	HOG + LBP + SVM	98.36	Not very useful for daily interact
2.2 Dynamic Sign words			
2.2.1 single-hand dynamic sign words recognition			
Naglot and Kulkarni [14]	LMC + MLP	96.15	Misclassification
Chopuk et al. [16]	LMC + polygon region + Decision tree	96.1	Misclassfication
Chong and Lee [15]	LMC + SVM + DNN	80.30 and 93.81	Occlusion and similarity
		Massey 99.39,	
Shin et al. [17]	SVM + GBM	ASL alphabet 87.60	error in estimated 3D coordinates
		FingerspellingA 98.45.	
Vaitkevicius et al. [37]	SOAP + MS SQL + HMC	86.1 ± 8.2	Misclassfication
Lee et al. [5]	LSTM + k-NN	99.44, at 5-fold 91.82	limited extensibility due to tracking
Chophuk and Kosin [31]	BiLSTM	96.07	limited extensibility to double hand
2.2.2 double-hand dynamic sign words recognition			
Igari and Fukumura [38]	minimum jerk trajectory +	98	Cumbersome + limited number
	DP-matching + Via-points + CC		of features
Dutta and GS [18]	Minimum EigenValue + COM	Text + Speech	Poor extensibility to word
	Server		
Demrcioglu et al. [19]	Heuristics + RF + MLP	99.03, 93.59, and 96.67	insufficient hand features
Deriche [20]	CyberGlove + SVM	99.6	Cumbersome + intrusive
DLMC-based ArSLRs [39]	LDA + GMM bayes classifier	94.63	sensor fusion complexity
			separate feature learning
Deriche et al. [40]	Dempster-Shaper + LDA + GMM	92	Misclassification and not mobile
Haque et al. [21]	Eigenmage + PCA + k-NN	77.8846	complex segmentation + few feature
Raghuveera et al. [22]	SURF + HOG + LBP +SVM	71.85	non-scalable features + segmentaton
Mittal et al. [30]	CNN + LSTM	Word 89.5	low accuracy due to weak learning
Katilmis and Karakuzu [41]	LDA + SVM + RF	93, 95 and 98	letters are not very useful for daily
			communication
Karaci et al. [23]	LR + k-NN + RF + DNN + ANN	cascade voting achieve	letters are not very useful for daily
		98.97	Fails to track double hands
Kam and Kose [25]	Expert systems + LMC + WinApp	SLR App	Mobility is not actualized
Katilmis and Karakuzu [24]	ELM + ML-KELM	96 and 99	complex feature extension may
			fall into over-fitting
Hisham and Hamouda [26]	Latte Panda + Ada-Boosting	double hand accuracy 93	similarity due to tracking issues
Avola et al. [32]	Multi-stacked LSTM	96	insufficient hand features

## 2. Materials and Methods

**Figure 1:**
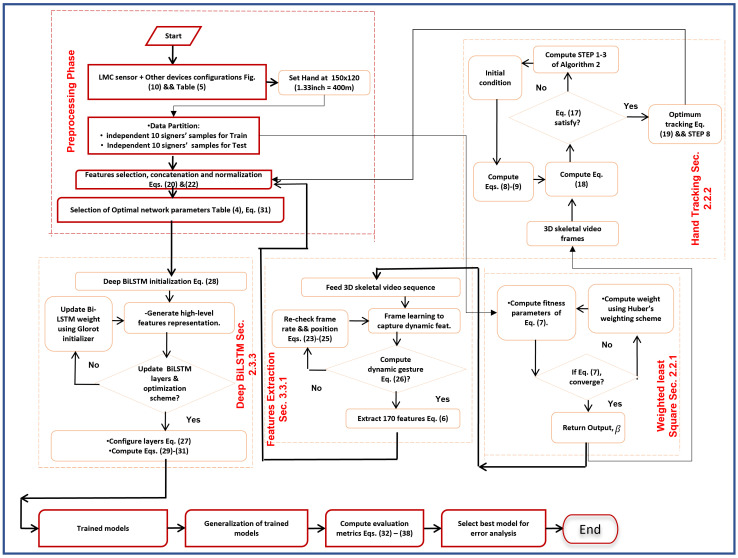
Procedures of the proposed method.

This section enumerates double-hand dynamic ASL words sign language recognition processes of the proposed method. We introduce our method in four subsections as follows: Section 2.2 skeletal video preprocessing, which encompasses the following: (a) weighted least square (WLS) algorithm for minimizing noise of 3D skeletal video sequence, (b) hand tracking using EKF method for tracking deep hand motion trajectories across video frames, (c) MIC for robust features selection, (d) features scaling to control hand dynamics and allow new signer, and (e) skeletal video frames correction to control initial frame coordinates and position of all consecutive frames. Section 2.3 ASL words recognition from skeletal video features encompasses the following stages: (a) bidirectional RNN (BiRNN) features extraction, (b) long short-term memory, and (c) multi-stacked deep BiLSTM training from transfer learning to learn temporal continuity of dynamic words. Section 2.4 encompasses model parameters. Section 2.5 encompasses evaluation metrics to calculate the overlap and similarity among the original dynamic ASL words and the predicted category videos for the recorded ASL words. Overall procedures of the adopted method are shown in Figure 1.

### 2.1. Basic Definition of Multi-Stacked Deep BiLSTM Feature

**Figure 2:**
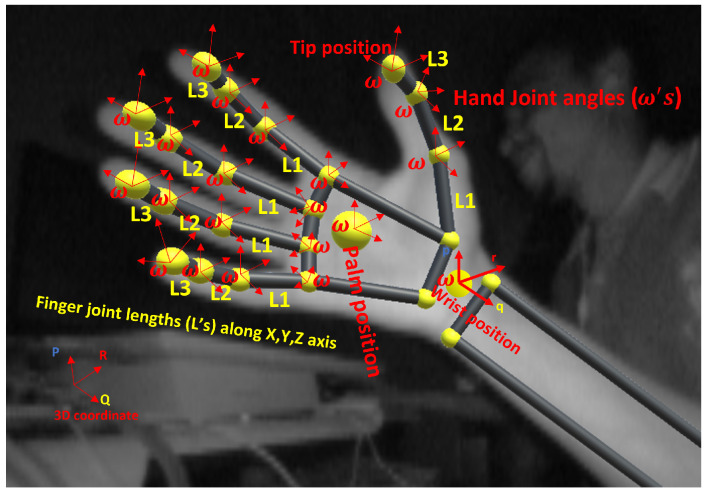
Skeletal palm joints with angle and joint length primes captured by LMC [43].

Sign language basic features (phonemes) [42] include hand shape, motion, orientation and location. (1) 3D dynamic hand shape characterizes double-hand dynamic ASL words, which can be obtained from the twenty-two skeletal hand joint primes *L* per each hand, thus, making a total of 44 primes for the double-hand $L_{44}$, along seventy angular features $\omega_{70}$ for the complete double-hand as described in Figure 2 and put in Equation (2). (2) 3D hand orientation provides angle coordinates at which the double-hands meet each other. The hand orientation angle is computed from seventy angle primes of seven major double-hand vertices, as described in Figure 2. However, hand location/position is obtained from direct measurement using LMC. Deep features are defined differently, but for the purpose of this article we have considered the following deep features. (1) Double-hand motion trajectories (MT), while performing ASL word, are defined as the action of two-hands in the LMC sensor’s field of view. This action is visualized as trajectory across video frames Equation (3) and can be tracked based on EKF algorithm. MT encodes correlation among hand movement and gesture dynamics. MT allow one to learn each dynamic across frames and to observe points where two gestures share similar characteristics, as mathematically established in Equation (3). Hand motion usually determines the frame speed of the video, which is coined in Equation (4). 3D dynamic hand motion is composed of velocity, which is comprises of action at beginning of gesture performance (preparation), peak acceleration (nucleus), and ending of gesture performance (retraction). Beginning and ending of gesture trajectory are known as preparation and retraction (that is, pause). (2) Hand Pause provides another potential information to discriminate similarity between dynamic gesture at the beginning or end of gesture characteristics. Thus, hand pause *P* is mathematically formulated within the leap motion Euclidean space in Equation (1). Significance of the proposed features to recognize double-hand ASL words is investigated using maximal information criterion (MIC) and cumulative match characteristics (CMC) curve.

(1) $$||P\left(t\right)||=\sqrt{P_{p}^{\left(2\right)}\left(t\right)+P_{q}^{\left(2\right)}\left(t\right)+P_{r}^{\left(2\right)}\left(t\right)}$$

where $P\left(t\right)=\left(p,q,r\right)\in R^{3}$.

(2) $$\begin{array}{c} \eta \left(p,q,r\right)=\left(L_{44}+\omega_{70}\right). \end{array}$$

However, for each dominant hand in video frame *f* at time *t*, while moving towards non-dominant hand (that is, the hands lined up to their orientation), hand motion trajectories across the consecutive frames at time *T*, can be expressed as ${\left[M_{t}^{\left(f\right)}\right]}_{t=1,\cdots ,T}$, where $M_{t}^{f}$ is defined as:

(3) $$\begin{array}{c} M_{t}^{\left(f\right)}\left(p,q,r\right)=\left(p_{t}^{\left(f\right)}+\sin\varphi_{t}^{\left(f\right)},q_{t}^{\left(f\right)}+\cos\varphi_{t}^{\left(f\right)},r_{t}^{\left(f\right)}+\tan\varphi_{t}^{\left(f\right)}\right)\in R^{3}. \end{array}$$

ASL word motion speed trajectory $K_{t}$ can be obtained at each fingertip. The fingertips provide hand motion in Equation (3), which can be formulated as follows:

(4) $$K_{t}\left(p,q,r\right)=\left|\right|k_{f}-k_{f-1}\left|\right|$$

where motion variation from *f*th to frame *f*th + 1 denotes speed difference and its correlation. With the addition of ($P_{t}^{f}$), ($\eta_{t}$) and ($K_{t}$) features, the functional Equation (6) can improve accuracy and reduce misclassification from double hand similar ASL words. Finally, the proposed features vector (β) of model [32] is defined by:

(5) $$\begin{array}{c} \beta_{t}\left(p,q,r\right)=\left[\omega_{t}^{f},N_{t}\right] \end{array}$$

To improve recognition accuracy and minimize misclassification of a set of double-hand dynamic ASL word feature vector, new features ($P_{t}^{f}$), ($\eta_{t}$) and ($K_{t}$), called basic and deep features, are added in Equation (5), and their functions are discussed in Equations (1)–(4), which can be uniformly written as:

(6) $$\begin{array}{c} \beta_{t}\left(p,q,r\right)=\left(P_{t}^{f}+\omega_{t}^{f}+\eta_{t}+N_{t}+K_{t}+\varphi_{t}\right) \end{array}$$

where ($P_{t}^{f}$), ($\omega_{t}^{f}$), ($\eta_{t}$), ($N_{t}$), ($K_{t}$) and ($\varphi_{t}$) denote pause, angles, shapes, positions (palm position displacement and fingertip position displacements), motion, and relative trajectory features in frame *f*th, at time *t*, respectively. Relative trajectory includes hand motion trajectories, speed, and relationship between dominant and non-dominant hand.

### 2.2. Skeletal Video Preprocessing

Noise such as large video frame sizes (due to large recording time) and human hand dynamics affects recognition performance of double-hand dynamic skeletal video information. The following sections employ robust tools to preserve the original video information free from noise.

#### 2.2.1. Weighted Least Square (WLS)

Skeletal video sequences are affected by noise (missing values), which has detrimental effect during recognition. This noise information is manifested among different video sequences, which influence the estimated original video sequence. To address this problem, WLS algorithm is chosen. WLS overcomes traditional drawback of linear regression, moving average and median filter problem of filtering only data sets with constant variance. WLS is a good choice of filter for many researchers in video processing [44,45,46,47,48]. Therefore, each sequence is allocated with suitable and effective weights to achieve significant confidence level with least residuals. The minimization of the errors in WLS is iteratively learned until weights of outliers are minuscule. The weights are obtained using Huber’s weighting scheme [44]. A given (*A*), ($A^{T}$), (*O*) and (*D*), which denote weight matrix, matrix transpose, response vector and diagonal matrix, contains weights associated with video samples; then, $\tilde{\beta }$ returns the estimate, as explained in Algorithm 1. WLS can be formulated as follows:

(7) $$\begin{array}{c} \left(A^{T}DA\left(w\right)\right)\lambda =A^{T}*D*O \end{array}$$

where *w*, *c*, β(w), $w_{f}$, *d(f)* and $l_{f}$ denote prediction time, order of prediction, raw video information, video progressing time, filter input and linear function. λ denotes wavelength parameter.
**Algorithm 1:** (*WLS*).***Input.** Set $l_{f}\left(w\right)={\left[100\cdots 0\right]}^{c}$, d(w)****Output.** WLS estimate $\tilde{\beta }=\left(A^{T}DA\left(w\right)\right)\lambda$****Step 1.** Compute $\beta \left(w\right)=l_{f}^{c}\left(w-1\right)d\left(w\right)$****Step 2.** Compute $l_{f}\left(w\right)$****Step 3.** Compute $A(w_{f})$ in Equation (7)****Step 4.** Update $A(w_{f})$ then****Step 5.** Finally we set w:=w+1 and return to step 1.*

#### 2.2.2. Hand Tracking Using Kalman Extended Filter (EKF)

**Figure 3:**
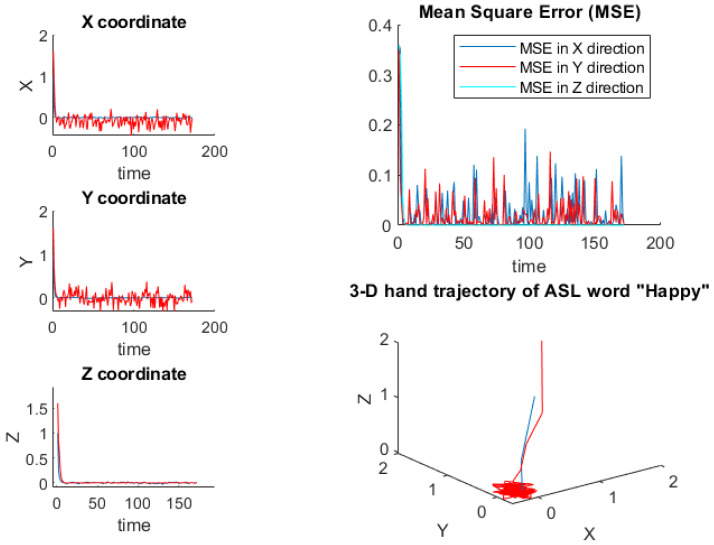
3D Hand motion trajectories across video frames using EKF.

The EKF is computationally efficient to our proposed data set, and the brief process is illustrated in flow chart Figure 1 and Algorithm 2. In each video frame, the two skeletal hands are learned from their registered starting point ($P_{t}$) to the hand resting point ($P_{t+1}$) while recording, as illustrated in Figure 3. The EKF involves estimating the process state with the aid of the equation of partial derivative and observation, using equation of partial derivative of process and observation, as shown in Equations (13) and (14). In Equation (13), $s\in R^{\iota }$, ϑ, $s_{c}$ and $\zeta_{c}$ denote nonlinear function, state variable, and process noise (feed back from LMC sensor). The nonlinear function evaluates the state according to the current moment *c*. The function parameters will extrapolate $g_{c-1}$ and $\zeta_{c}$. In Equation (14), $d\in R^{\tau }$, $d_{c}$, ϕ and $\Omega_{c}$ denote observed variable, nonlinear function and observation noise (feed back from LMC sensor). Therefore, to incorporate the process of a nonlinear difference and observation noise for real-life usage, modified Equations (13) and (14) are adopted from [49]:

(8) $$\begin{array}{c} s_{c}\approx \tilde{s_{c}}+I\left(s_{c-1}-{\hat{s}}_{c-1}\right)+\zeta \zeta_{c-1} \end{array}$$

where $s_{c}$, *I*, $\tilde{s_{c}}$ and ${\hat{s}}_{c-1}$ denote original information of the state vector, Jacobian matrix of the partial derivative of ϑ with respect to *s*, observation information of the state vector, and state vector posteriori probability of moment *c*.

(9) $$\begin{array}{c} d_{c}\approx \tilde{d_{c}}+U\left(s_{c}-{\tilde{s}}_{c}\right)+\Omega \Omega_{c} \end{array}$$

where $d_{c}$, *U* and $\tilde{d_{c}}$ denote original information of the observation vector, Jacobian matrix of the partial derivative of ϕ with respect to Ω, and observation information of the state vector. The Jacobian cannot be estimated mathematically with noise term; therefore, it is assumed as zero. Thus, the Jacobian matrices can be obtained as follows:

(10) $$\begin{array}{c} I_{n,f}=\frac{\partial \vartheta_{n}}{\partial s_{f}}\left\{{\hat{s}}_{c-1},g_{c-1},0\right\} \end{array}$$

(11) $$\begin{array}{c} U_{n,f}=\frac{\partial \Omega_{n}}{\partial s_{f}}\left\{{\tilde{s}}_{c},0\right\}. \end{array}$$

However, the residuals of the observed variables and prediction error can be obtained from the covariance matrix in Equation (19).

The covariance matrix is independent from random variables that provide an approximation using Equations (13) and (14). From this approximation, the EKF can be extended to estimate the equation, thus

(12) $$\begin{array}{c} {\hat{s}}_{c}={\tilde{s}}_{c}+C_{c}{\tilde{r}}_{d_{c}}={\tilde{s}}_{c}+C_{c}\left(d_{c}-{\tilde{d}}_{c}\right). \end{array}$$

Finally, Equation (12) is utilized to adopt the observation variables of EKF. ${\hat{s}}_{c}$ and ${\tilde{d}}_{c}$ can be obtained from Equations (13) and (14), respectively. From the results in Figure 3, we have the following observations. (1) Blue plot indicates the original 3D hand motion trajectory along with its corresponding mean square error (MSE). Red plot indicates the estimated 3D hand motion trajectory along with its corresponding MSE. Individual axis performance of EKF algorithm is demonstrated by the left plots. EKF algorithm achieves very competitive tracking across the 3-axis by observing the MSE, which validates the stability of EKF algorithm for complete hand motion trajectory. (2) As the ambiguity/uncertainty rate increases, the performance degradation (high MSE) of the compared original measurements is much larger than that of EKF tracking.
**Algorithm 2:** (EKF).***Input.** Choose any arbitrary actual initial conditions w, initial observations m, assumed **initial conditions j, covariance of estimation initial value h, the sampling time t, indx = 0,** and n = 1:170.****Initial setting.** Let $d_{c}$, $s_{c}$, h and S be covariance matrix of process noise, measurement** noise, estimation error and original information.****Output.** 3D EKF estimate $\tilde{M_{n}}$.****Step 1.** Determine process and observation along X, Y, and Z coordinates, from*(13)$$s_{c}=\vartheta \left\{s_{c-1},g_{c-1},\zeta_{c-1}\right\}$$(14)$$d_{c}\in \phi \left\{s_{c},\Omega_{c}\right\}$$***Step 2.** Compute prediction function*(15)$$j\left(n\right),h\left(n\right):=predict(S,j\left(n\right),h\left(n\right),d_{c}).$$***Step 3.** Compute Jacobian matrices in Equations (10) and (11).****Step 4.** Computes Kalman gain*(16)EKF(n+1)=Gain(H(n+1),P(n+1),M)***Step 5.** Compute overall estimate*(17)j(n+1)=j(n+1)+EKF(n+1)*G*where****Step 6.** G is the filter specialty, estimates from*(18)G=G(m(n+1),j(n+1),indx)***Step 7.** Compute covariance estimation error*(19)$${\hat{s}}_{c}={\tilde{s}}_{c}+{\hat{r}}_{c}$$***Step 8.** Compute MSE along X, Y, and Z. as shown in Figure 3****Step 9.** Finally, we set n:=n+1 and return to step 1.*

#### 2.2.3. Maximal Information Correlation (MIC)

**Figure 4:**
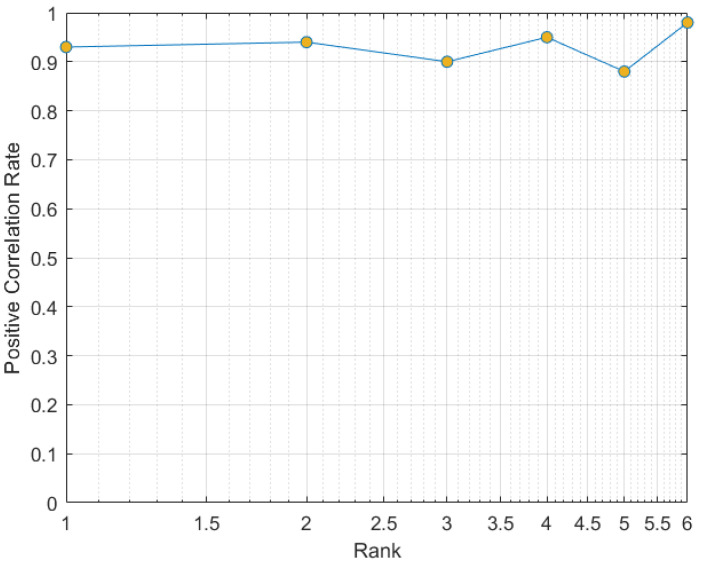
Cumulative match characteristics curve of the features from MIC.

We introduced a feature selection method derived from correlation analysis known as MIC to reduce the complexity of the deep learning algorithms. MIC utilizes 3D video features between zero and one. The significance of adopting MIC was the capacity to treat nonlinear and linear unions among video data sets. It makes no assumptions about the distribution of the recorded video. However, MIC has simple computing formula, and it applies to sample sizes *t* ≥ 2. MIC of 3D vectors *p*, *q* and *r* is defined as follows [50,51], and the results are displays in Table 2:

(20) $$MIC=max\{\frac{I(p,q,r)}{{log}_{2}min\left(t_{p},t_{q},t_{r}\right)}\}$$

where

(21) $$\begin{array}{cc} I(p,q,r) & =H\left(p\right)+H\left(q\right)+H\left(r\right)-H\left(p,q,r\right)=\sum_{u=1}^{t_{p}}p\left(p_{u}\right){log}_{2}\frac{1}{p(p_{u})}+\sum_{v=1}^{t_{q}}p\left(q_{v}\right){log}_{2}\frac{1}{p(q_{v})}\\ \; & \;\;+\sum_{w=1}^{t_{r}}p\left(r_{w}\right){log}_{2}\frac{1}{p(r_{w})}-\sum_{u=1}^{t_{p}}\sum_{v=1}^{t_{q}}\sum_{w=1}^{t_{r}}\;p\left(p_{u},q_{v},r_{w}\right){log}_{2}\frac{1}{p(p_{u},q_{v},r_{w})} \end{array}$$

where *p*, *q* and *r* denote feature vectors along 3D axis. *H*; *I*; *B*; and $p_{u}$, $q_{v}$ and $r_{w}$ denote entropy, information, bins and number of bins of the partition along 3D axis. Note: $p_{u}.q_{v}.r_{w}<B\left(t\right)$ and $B\left(t\right)=t^{\left(0.6\right)}$. The MIC analysis demonstrates the effectiveness of the proposed features as shown in Table 2. In Table 2, the diagonal values indicate correlation of each feature with itself, while all other values inside the table indicate the correlation of each feature against it neighbor. Positions having values ranges 0.9 to 1 are regarded as having strong correlation, whereas values less than 0.9 are still significant and are conserved during model design. All other features less than 0.8 were disregarded in this paper. Furthermore, we investigated the significance of the selected features according to the cumulative match characteristics curve (CMC), as illustrated in Figure 4. The CMC plot is generally used to quantify the correlation between detection rate and the rank score from the given features. We evaluated different feature combinations across all the features, but the following were found to be effective according to CMC ranking: 1st (shape + position + motion), 2nd (shape + position + angle), 3rd (shape + position + motion + angle), 4th (shape + angle), 5th (shape + position + motion + angle + pause + relative trajectory) and 6th (shape + position). In this plot, each features combination exempted the knowledge of hand dynamics (pause and relative trajectory), while the remaining features were evaluated so that measure of the contribution of our added feature per each combinations was obtained. Thus, best feature combinations were achieved with least score at 5th rank (shape + positions + motion + angles + pause + relative trajectory features), whereas less significant features combination was achieved with high score from the 6th rank (shape + position features), as shown in Figure 4. Therefore, it is difficult to achieve best recognition with features combination, due to absence of hand dynamics knowledge.

#### 2.2.4. Features Scaling

*Z*-score transformation is applied to scale independent features at each video frames at some threshold range. Feature scaling is carried out due to learning network employed gradient descent, which converges faster than non-scaled features. Z-score transforms each feature information from zero to its unit variance. Thus, Z-score is given by

(22) $$Z-transform=\frac{\left(\beta -mean(\beta )\right)}{s.t.d(\beta )}$$

#### 2.2.5. Skeletal Video Frames Correction

We use the video frame manipulation (correction) strategy to control initial frame coordinates and position. This is because of the different hand speeds and variations (intuitive interaction) during dynamic word performance. We address this is to highlight the subsequent frame in the sequence, when two or more gestures exhibit different hand trajectories [52]. In what follows, we exploit information of all the frames in the sequence. From each sequence, we calculate the average distance among the frames at $F_{P}$, $F_{Q}$ and $F_{R}$. The average distance is considered for each feature value, which can be utilized to correct the video frames. The technique is mathematically coined as follows:

(23) $$F_{P}=\frac{\sum_{t=1}^{170}\left(ValidationSet\beta_{t,Q}-TrainingSet\beta_{t,P}\right)}{170}$$

(24) $$F_{Q}=\frac{\sum_{t=1}^{170}\left(ValidationSet\beta_{t,Q}-TrainingSet\beta_{t,Q}\right)}{170}$$

(25) $$F_{R}=\frac{\sum_{t=1}^{170}\left(ValidationSet\beta_{t,R}-TrainingSet\beta_{t,R}\right)}{170}$$

where validationSet, TrainingSet, $\beta_{t}$ and *t* denote testing information (along *P*, *Q* and *R*), training information (along P, Q and R), feature vector, and amount of video frames (t=1,⋯,170), respectively. This is done by subtracting the first thirty sequences in the feature vector. The three equations make the initial position of each trajectory per frame similar to the frame coordinates. This allows us to compute each dynamic across frames.

### 2.3. ASL Word Recognition from Skeletal Video Features

**Figure 5:**
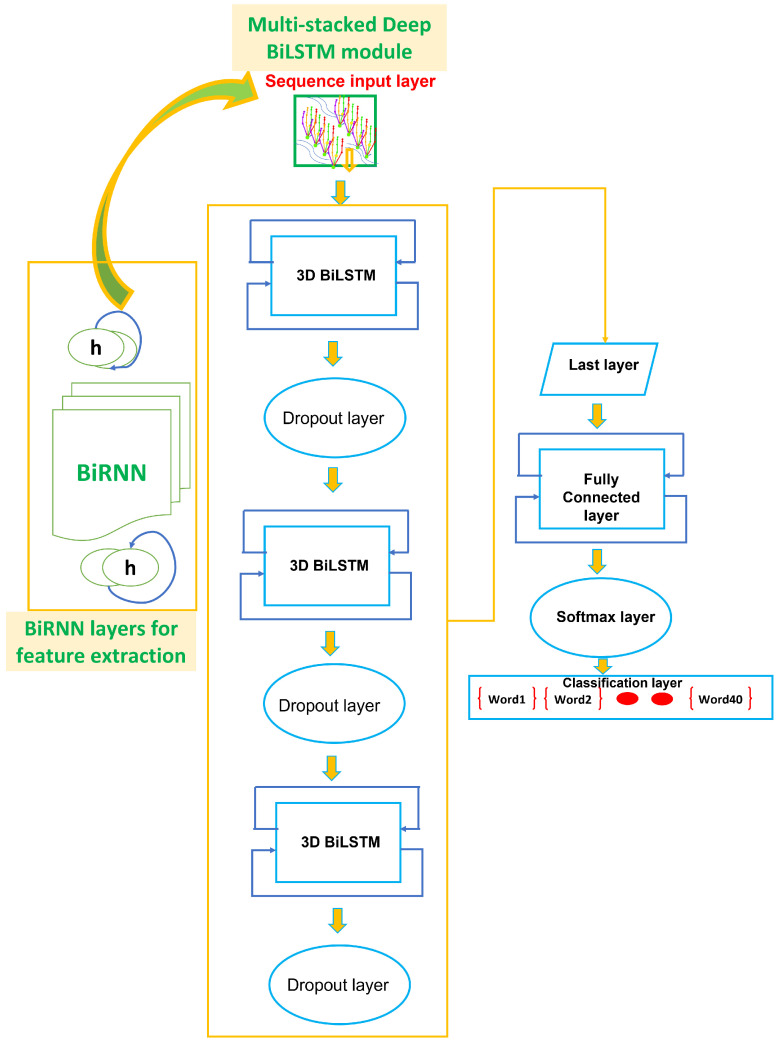
Proposed Architecture of Multi-stack Deep BiLSTM.

The double-hand dynamic ASL word-recognition system is illustrated in Figure 5, which is comprised of the two modules: BiRNN and multi-stacked bidirectional-LSTM.

#### 2.3.1. BiRNN Features Extraction

Skeletal joints are automatically extracted using bidirectional recurrent neural network (BiRNN). Empowering the RNN architecture with two BiRNN layers improves the learning behavior with symmetrical, previous and subsequent frame for each information in the video sequence [53] and no re-positioning of the input videos from the ground truth or intended sequence. Nonlinear operations and architecture with hidden layers of BiRNN allow one to find patterns in video sequence. BiRNN is designed and trained using multi-stacked layers in two fashions to extract hand features from skeletal video. We recorded hand gesture video information $v_{n}$ from input video frame $Q_{f}$ with sequence length Ω. This input video sequence was fed to BiRNN layer. $v_{n}$ is defined as ($v\in Q_{f}$) where 1≤*n*≤Ω. BiRNN layers received input video sequence $Q_{f}$, and th function in Equation (26) was evaluated to update its *n*-hidden states, according to the input units $\left[h_{1},h_{2},\cdots ,Q_{t}\right]$, until it learned the last hand gesture video information in the last video frames $v_{n}=0$. The information in the present layer is automatically opposite to the hidden units (layers), so the output layer will not update till the hidden units have processed the whole video information. For the backward direction, the total output layer units are computed, and fed back into the hidden layers in opposite directions. The second phase of the BiRNN layers is trained to learn output of the previous layers to be initial state of first layers and yields output vector $\beta_{t}=[t_{1},\cdots ,t_{\Omega }$], and it is defined as: $t_{\Omega }\in \beta_{t}$, where 1≤*n*≤Ω. Finally, BiRNN extraction layers can be written uniformly as [43]:

(26) $$h_{t}=\sigma \left[V_{\vec{hq}},Q_{f,n}+V_{\overset{\leftarrow }{hq}},Q_{f,n}+V_{\vec{hh}},Q_{f-1,n}+V_{\overset{\leftarrow }{hh}},Q_{f-1,n}+d_{h}\right].$$

where dominant and non-dominant hand index is denoted as *n*, and ($\vec{h_{q}}$) and $\overset{\leftarrow }{h_{q}}$ denote forward and backward pass hidden state vectors, respectively. In Equation (26), the extraction layers of BiRNN not only give the relationship of video input features vector but also correlate to state of prior sequence.

Moreover, after extracting the matrices of the six selected features, we transformed the matrices into a feature vector. However, many techniques are available for feature transformation such as columns concatenation, rows concatenation and zigzag scheme. As reported in the literature rows, concatenation demonstrates best concatenation. Thus, we convert matrix into feature vector to obtain features in Equation (6). Equation (6) provides training input sequence (six proposed extracted features). The 3D skeletal hand joints are extracted and represented as input features vector, as illustrated in Table 3.

#### 2.3.2. Long-Short Term Memory (LSTM)

LSTM is a family of RNN to handle gradient vanishing, by substituting an extended bidirectional LSTM (BiLSTM) neurons [27,54,55]. BiLSTM neuron learn long-term dependencies between sequences [5,31,56]. Single BiLSTM unit return low accuracy especially when learning complex sequences. Deep BiLSTM is introduced to enhance accuracy of single LSTM unit. Multiple long short-term memory (known as deep BiLSTM) architecture is the strategy of concatenating number of BiLSTM hidden units in fashionable manner. This is to achieve high-level sequential representations from sequential video information. In deep BiLSTM, output of former layer *l-*1 serves as sequence input to present layer *l*. Results demonstrated that deeper networks improve recognition performance [36].

Deep BiLSTM network is illustrated in Figure 5, which is realized by concatenating three-additional BiLSTM layers with output mode “sequence” before each BiLSTM layer. Dropout layer is connected after each BiLSTM layer to control overfitting and alter fundamental network architecture, which is defined in Equation (27) [57]. The final output of all sequences is concatenated to construct one output layer known as softmax layer. The output mode of last BiLSTM layer is now coined as “last”. Therefore, ASL words class prediction is conducted by equipping the last layer of BiLSTM network with classification layer. Classification layer is configured with cross entropy loss function [58]. The fully connected layer multiplies sequential input by weight α and then adds ρ. However, fully connected layer merges all features in $\beta_{t}$ to classify word gesture. In our case, information from fully connected layer of deep multi-stack BiLSTM network is exactly the same as the number of word classes of sequential features. This procedure is known as multi-stacking, and the architecture is referred to as multi-stack deep BiLSTM.

(27) $$rand(size\left(d_{i}\right)<probability$$

where $d_{i}$ denotes drop layer input.

#### 2.3.3. Multi-Stacked Deep BiLSTM Training from Transfer Learning

**Figure 6:**
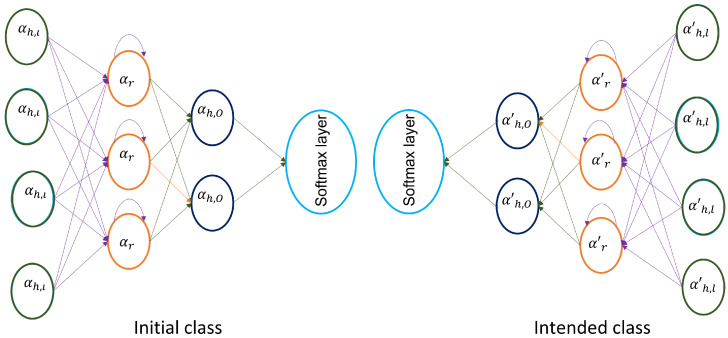
Diagram of transfer deep LSTM network.

The major limitation of training multi-stacked deep BiLSTM network is the high demand for large input video set. The number of our input video sets is moderate. However, training a new BiLSTM network is a complex and costly process. Multi-BiLSTM network from the existing method has large number of abstractions, and this makes learning difficult. This can lead to misclassification. To overcome this problem, transfer learning (TL) via deep neural network is extended to SLR. TL is a methodology of utilizing a pretrained deep network that has proven successful as initial step (newly designed network) to learn feature from unknown signer. A methodology of fine-tuning network brings simple and fast learning network, compared to conventional network initialized from the grass-root. Researchers identified the potential of neural-network-based TL [59,60]. In this paper, TL approach based on multi-stack deep BiLSTM network as shown in Figure 6 is implemented to recognize dynamic double-hand ASL words. Extracted input feature vector in Equation (6) is built into multi-stacked BiLSTM layers for double-hand dynamic ASL words recognition. Multi-stacked BiLSTM is trained to obtain output probability vectors for all of its corresponding input vectors, predicted word classes, and confusion matrices. Multi-stacked layers are initialized with weight of extracted features, as follows [43]:

(28) $$\begin{array}{cc} O_{t,n} & =[V_{\vec{h}o}{\vec{h}}_{f},P_{t,n}^{f}+V_{\overset{\leftarrow }{h}o}{\overset{\leftarrow }{h}}_{f},P_{t,n}^{f}+V_{\vec{h}o}{\vec{h}}_{f},\omega_{t,n}^{f}+\\ \; & \;\;+V_{\overset{\leftarrow }{h}o}{\overset{\leftarrow }{h}}_{f},\omega_{t,n}^{f}+V_{\vec{h}o}{\vec{h}}_{f},\eta_{t,n}+\\ \; & \;\;+V_{\overset{\leftarrow }{h}o}{\overset{\leftarrow }{h}}_{f},\eta_{t,n}+V_{\vec{h}o}{\vec{h}}_{f},N_{t,n}+V_{\overset{\leftarrow }{h}o}{\overset{\leftarrow }{h}}_{f},N_{t,n}+\\ \; & \;\;+V_{\vec{h}o}{\vec{h}}_{f},K_{t,n}+V_{\overset{\leftarrow }{h}o}{\overset{\leftarrow }{h}}_{f},K_{t,n}+\\ \; & \;\;+V_{\vec{h}o}{\vec{h}}_{f},\varphi_{t,n}+\\ \; & \;\;+V_{\overset{\leftarrow }{h}o}{\overset{\leftarrow }{h}}_{f},\varphi_{t,n}+d_{o}]. \end{array}$$

The final classification layer is formulated as follows:

(29) $$\begin{array}{c} O=\sum_{\Omega =0}^{\Omega -1}O_{\Omega ,n}^{t} \end{array}$$

(30) $$\begin{array}{c} O^{t}=p\left(E_{L}|\beta \right)=\frac{e^{O^{L}}}{\sum_{i=0}^{L-1}e^{O_{i}}},L=1,\cdots ,L \end{array}$$

where *L* and $O_{\Omega }^{t}$ denote ASL word classes and predicted probability class $E_{L}$ when ASL word features β is given, respectively. However, softmax function transforms the output value into [0, 1] and transforms the weight of *L* values into 1. The ground truth is given as $O^{L}\in \left[0,1\right]$, as well as prediction probabilities as $\vec{O^{L}}$. The network parameters can be given as in Equation (31), as follows:

(31) $$\begin{array}{c} \theta \left[u+1\right]=\theta \left[u\right]+r\left(-\frac{\partial O}{\partial \theta [u]}\right),\theta \left[0\right]\approx U\left[0,1\right]. \end{array}$$

From Equation (31), θ[u], *u* and *r* denote parameters set, parameter update times, and learning rate. This equation consists of all weights and biases in the Deep BiLSTM network.

Let an initial class $C_{i}=\beta_{i}$ have a learning period $p_{i}$; thus, the intended class $C_{d}=\beta_{d}$ has a learning period $p_{d}$. Thus, the aim is to aid learning the prediction function of the intended class by utilizing knowledge gained by $p_{i}$ from initial class. However, transfer learning has a rule: that the initial class should be different from the intended class, as well as their learning periods. For the intended class, we have recorded 40 ASL words from 10 signers, which are repeated 10 times, making a total of 4000 samples, whereas for the initial class, we have recorded 10 signers different from the ones in the intended class; however, each signer performs 58 ASL words, 10 times, which makes a total of 58,000 sequences. For details of the experimental set up and data recording process, see Section 2.6.1.

The feature learning phase of the network has five layers, as illustrated in Figure 5. In Figure 6, the features of a successful network can be reused in a newly adopted network. The weight matrices among input and the hidden layers $\alpha_{h,l}$, recurrent weight matrices in the hidden layers $\alpha_{r}$, and connection weight matrices among hidden layers and output layer $\alpha_{h,o}$ were trained in the initial class (trained in advance with sufficient features). The successful network is illustrated in Figure 5. Thus, the weight matrices among input layers and hidden layers are transferred to intended class features as weight initial value. This new approach of weight initialization is superior to random initialization. However, training features of intended class were used to adjust the BiLSTM weights on small data set. Thus, recurrent weight matrices in the hidden layers, and connection weight matrices among hidden layers and output layer, were initialized at random.

### 2.4. Model Parameters

The selected method is experimentally validated with careful selection of parameters in Table 4. These parameters were achieved through cross-validation. Our experiments are designed from personal computer (PC) on Windows 10 operating system equipped with CPU Core i7 9th Gen, 8 GB RAM, details of the execution environment is provided in Table 5. Serial communication from LMC to PC is enabled via written C# codes on Microsoft visual studio environment, and LabView library.

### 2.5. Evaluation Metrics

Confusion matrix contains columns and rows, where each column denotes possibility of predicted word gestures, whereas each row denotes original word gesture probability. However, main diagonal of confusion matrix denotes scores of correct classified word gestures with blue colors, whereas entries below diagonal denote false positives (gestures classified incorrect from our concerned class) with gold color, and entries above diagonal denote false negatives (gestures classified incorrect from non-concerned class) with dark orange color. From confusion matrix, set of word pairs inside similar cell and in similar class is denoted as true positive,$\tau_{1}$; set of word pairs inside similar cell and in different class is denoted as true negative, $\tau_{2}$; set of word pairs inside different cell and in different class is denoted false positive, $\psi_{1}$; and set of word pairs in different cells and in different classes is denoted false negative, $\psi_{2}$. Each word pair is computed based on its frequency of occurrences. However, it is demonstrated that $\tau_{1}$ and $\psi_{2}$ should be maximized and $\tau_{2}$ and $\psi_{1}$ minimized to better explore performance of selected features and to determine optimal multi-stacked deep BiLSTM recognition. The following metrics are most popular for deep neural network and provide the results of comparison [5].

#### 2.5.1. Accuracy Metrics

*Accuracy* is described as measure of correct predictions. *Accuracy* is given by:

(32) $$Accuracy=\frac{\tau_{1}+\tau_{2}}{\tau_{1}+\tau_{2}+\psi_{1}+\psi_{2}}$$

Furthermore, accuracy index is not resourceful when two word classes are of varied sizes; this leads one to obtain high measure of correct predictions. To overcome this daunting problem, the following indices are augmented as best choices [61]:

#### 2.5.2. Fowlkes–Mallows (*FI*) Index

Fowlkes–Mallows index is adopted to evaluate level of similarity between trained and predicted word classes.

(33) $$FI=\sqrt{\frac{\tau_{1}}{\tau_{1}+\tau_{2}}*\frac{\tau_{1}}{\tau_{1}+\psi_{1}}}$$

#### 2.5.3. Matthew Correlation Coefficient (*MC*)

Matthew correlation coefficient determines trained and predicted binary classification [62], which is defined as:

(34) $$\begin{array}{c} MC=\frac{\left(\tau_{1}*\tau_{2}\right)-\left(\psi_{1}*\psi_{2}\right)}{\sqrt{\left(\tau_{1}+\psi_{1}\right)\left(\tau_{1}+\psi_{2}\right)\left(\tau_{2}+\psi_{1}\right)\left(\tau_{2}+\psi_{2}\right)}} \end{array}$$

#### 2.5.4. Sensitivity $\left(S_{v}\right)$

Sensitivity is defined as:

(35) $$\begin{array}{c} S_{v}=\frac{\tau_{1}}{\left(\tau_{1}+\psi_{2}\right)} \end{array}$$

#### 2.5.5. Specificity $\left(S_{f}\right)$

Specificity is defined as:

(36) $$\begin{array}{c} S_{f}=\frac{\tau_{2}}{\left(\tau_{2}+\psi_{1}\right)} \end{array}$$

#### 2.5.6. Bookmaker Informedness (*BI*)

Bookmaker informedness determines probability estimate of informed decision; it is defined as:

(37) $$\begin{array}{c} BI=(S_{v}+S_{f})-1 \end{array}$$

#### 2.5.7. Jaccard Similarity Index (*JI*)

*JI* metrics describes portion of overlap between two words: word 1 (trained word) and word 2 (generalized word), where they share similar features. These features are considered 0 or 1. Each feature per particular class must fall into one of $\tau_{1}$, $\psi_{2}$, $\tau_{2}$ and $\psi_{1}$ entries, respectively. *JI* is given as [63]:

(38) $$\begin{array}{c} JI=\frac{\tau_{1}}{\left(\tau_{1}+\tau_{2}+\psi_{1}\right)} \end{array}$$

Moreover, the developed models from the proposed method are evaluated using the accuracy, sensitivity, and specificity metrics. However, the best model is subject to further evaluation using K-fold and LOSO cross-validation to observe the influence of majority over the minority classes (class imbalance). The shortcomings of these recognition metrics include displaying misguiding results on imbalanced features due to failure to accommodate the relationship between the positive and negative entries in the confusion matrix. In addition, these metrics were not good enough to evaluate the matrix overlap. Therefore, to monitor the exact classification accuracy of our best model and to overcome the limitations of the mentioned metrics, we extend the evaluation of *MC*, *JI*, *BI* and *FI* indices. These metrics were reported in some studies to demonstrate good performance.

### 2.6. Experiment

**Figure 7:**
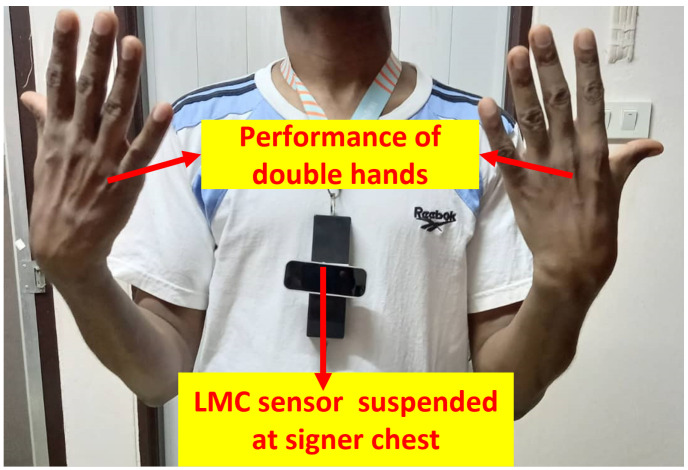
Experimental set up.

In this section, we present the experimental procedures of the implemented system. The system is implemented using best hardware selection details on Table 5, which are assembled to provide the experimental set up of Figure 7. In the simulation task, several Matlab packages were used to validates the network performance.

#### 2.6.1. Dataset Design

Available public hand skeletal ASL datasets with resourceful 3D skeletal hand information while signer is on the move, as in our proposal, are scanty, thus making it necessary to construct our data sets. In this approach, we selected 40 dynamic double-hand ASL words from first 200 available ASL words vocabulary. All signs were captured from 10 right-handed (right hand as dominant hand) double-hand signers. We extended strategy for in-the-field data design in [27]. All signers were trained from web ASL video information tutors. Age range of signers was 25–40 years. Each signer repeated double-hand ASL word 10 times, making a total of 4000 samples. LMC is suspended on signer’s chest, as shown in Figure 7, to actualize ubiquitous sign language recognition system. LMC is a vision-based capturing devices that employs infrared image sensing analogy at 30 frame per second, with 2×640×240 range to extract 3D hand-joint skeletal video information. LMC SDK software is configured via API (application programming interface) to synchronize with MS visual studio and LabView frameworks for data recording and visualization. Brief description of our designed data set is details in Table 6. We recorded 170 frames per each 131 skeletal video sequence length. However, some video frames contained sequence length less than 131. Then, we applied padding procedures in [32] to obtain equal number of sequence length. Our adopted network was further validated on the three challenging public-hand skeletal dynamic gestures from LMC data bases as follows. These data sets were evaluated according to the leave-one-subject-out experimental protocol:

- Avola et al. [32] data set: the data set is comprised of static and dynamic skeletal hand gestures captured from 20 signers, and it is repeated twice. Due to the nature of our approach, we selected dynamic gestures, including bathroom, blue, finish, green, hungry, milk, past, pig, store and where.
- LMDHG [64] data set: comprised of dynamic skeletal hand gestures collected from 21 signers, each signer performed at least one sign, resulting in 13 ± 1 words.
- Shape Retrieval Contest (SHREC) [65] data set: Comprised of 14 and 28 challenging dynamic skeletal hand gestures, respectively. The gestures were performed using one finger and the entire hand.

## 3. Results

In this section, we present simulation results of the adopted multi-stacked deep BiLSTM networks. Two type of deep networks were designed and simulated to demonstrate accuracy of our selected features, as shown in Table 7.

**Figure 8:**
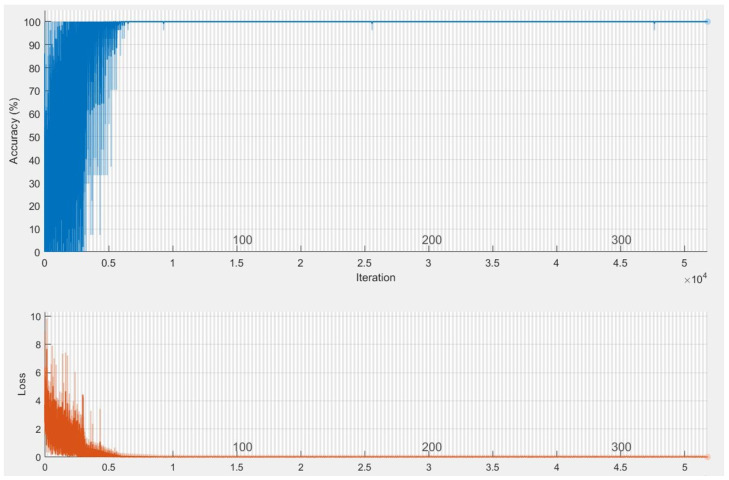
Training performance of Deep BiLSTM network on Model-1.

**Figure 9:**
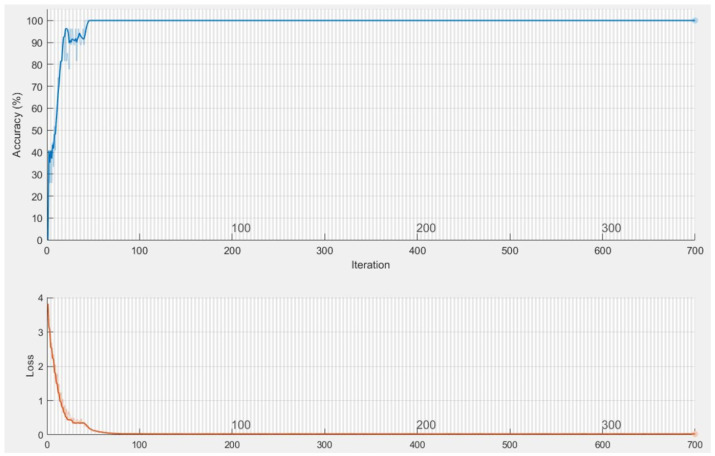
Training performance of Deep BiLSTM network on Model-2.

**Figure 10:**
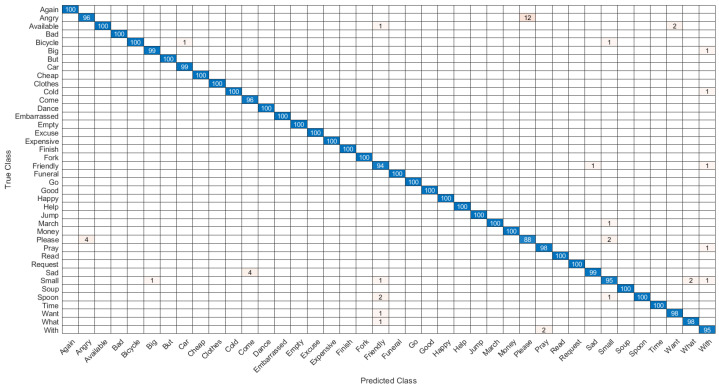
Confusion matrix of the recognition performance of double-hand dynamic ASL words with Adam optimization.

**Figure 11:**
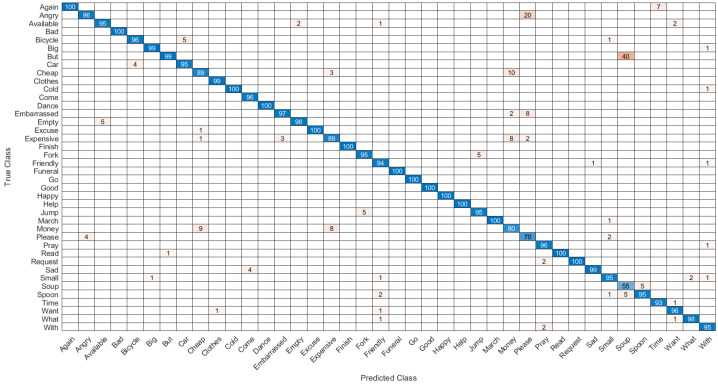
Confusion matrix of the recognition performance of double-hand dynamic ASL words with SGD optimization.

**Figure 12:**
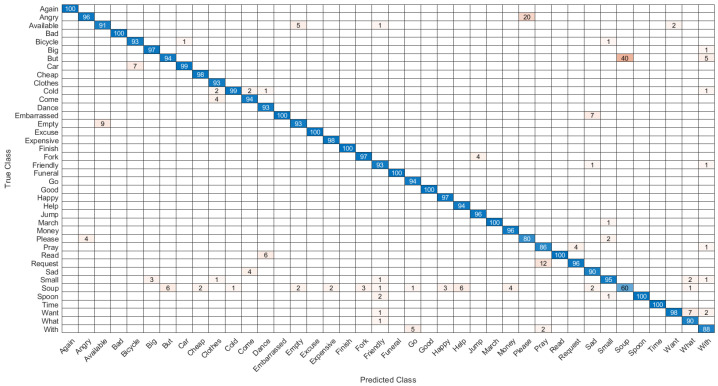
Confusion matrix of the recognition performance of double-hand dynamic ASL words with Adagrad optimization.

The first network combined hand shape, motion, position, pause, angle and relative trajectory. After several trial and error parameter selections, it was found that best model for different input feature combinations settled at Model-1 with 3 multi-stacked deep BiLSTM layers. Model-1 was trained at 300 epochs, where each class pair had inferences at 1.05 s, as illustrated in Figure 8 and Table 7. The second network was made through combination of shape, motion, position, and angle features. After several trials of network training for different feature combinations, best model was settled at Model-2 with 3 multi-stacked deep BiLSTM units, inferences at 1.01 s via 300 epochs, as illustrated in Figure 9 and Table 7. Since Model-1 achieved best recognition, we subjected it to further analysis using Leave-One-Subject-Out (LOSO) protocol because of its robustness, where 9 signers out of 10 were trained and the remaining signer was used during validation (generalization). This procedure was repeated 10 times, and the results are reported in Table 8. We achieved best LOSO validation due to reduced cost from the TL. Good discrimination performance was noticed by the developed multi-stacked deep BiLSTM when knowledge of hand dynamics were used in the input vector, achieving average sensitivity of 97.494%, specificity of 96.765%, average *FI* of 72.347%, *MC* of 94.937%, *BI* of 94.259%, *JI* of 54.476% and accuracy rate up to 97.983%. Therefore, the two models were set to inference with Top-K validation [66]. The data set was partitioned into 80% and 20% for training and validation, respectively. In this trial, K took values of 1, 2 and 3. Results of Top-3 validation are demonstrated in Table 9. It is demonstrated that model-1 achieved best accuracy of the three classes (*k* = 1, 2, 3). This indicates that additional feature from pause and relative trajectory (knowledge of hand dynamics with motion speed) contributed to 4% accuracy when compared to second model with only four input features. Table 10 summarizes the computing cost required to test our best model. An ablation investigation of our designed data set revealed the influence of stacking multiple BiLSTM layers. The multi-stacked BiLSTM network was trained using the three network performance schemes to optimize the loss function. Figure 10, Figure 11 and Figure 12 demonstrate the recognition performance of multi-stacked deep BiLSTM network with optimization from Adam, stochastic gradient descent and adaptive gradient schemes, respectively. Their performance comparison of computed mean of standard evaluation metrics is displayed in Table 11, which is obtained by condensing the entire confusion matrix for the average results. The best optimization scheme for multi-stacked deep BiLSTM with Model-1 input feature vector is Adam.

Table 12 provides performance comparison between average recognition accuracy of Model-1 and proposed method in [32]. The work [32] has similar shape with our approach because this method utilized gestures from ASL dictionary. Their method employed 20 signers, and each signer was directed to perform 12 dynamic double- and single-hand ASL words, 30 times each. Our multi-stack deep BiLSTM network was outperformed [32] on ASL data set with accuracy, precision, recall and F1-Score of 1.48%, 1.597%, 1.469%, and 1.626%, respectively. These results are consistent with the skeletal dynamic hand gesture recognition. When our method was validated on LMDHG data set, it was outperformed [32] with mild recognition accuracy of 0.37%. In addition, our method was validated on SHREC data set, and work in [32] was superior to our technique by 0.63% for experiment with 14 hand gestures, while we outperformed [32] by 1.56% for experiment with 28 hand gestures.

### Performance Comparison with Baseline Methods

Validation is carried out with various baselines on the LMDHG and SHREC’17 databases, respectively. Different results are illustrated and analyzed.

In Table 13, evaluation results of SHREC’17 dataset from standard protocol are illustrated. Methods Avola et al. [32] and Li et al. [67] are in similar shape with our approach, and their results are obtained from [65,68]. In particular, our method obtains 96.99% on the 14-gesture protocol and 92.99% on the 28-gesture protocol. It outperforms the most recent works [67,68] by 0.48% and 0.68% for experiment with 14 hand gestures and by 1.5% in [68] for experiments with 28 hand gestures, respectively, though [67] is superior to our technique by 0.34% for experiment with 28 gestures. However, our method demonstrates state-of-the-art performance on recent approaches. Table 14 illustrates evaluation results of LMDHG data set. The comparison results are obtained from works in [32,64]. Our method outperforms the two recent approaches in [26,35] on LMDHG data set with average recognition accuracies of 6.79% and 5.99%.

## 4. Discussion

We combined two set of models from two different input feature set combination to improve hand feature recognition and examine sensitivity per features against recognition accuracy. Sometimes, true and false negatives revealed zero values (with best true positive), and evaluating these values using standard metrics produced a misleading conclusion. Therefore, for better explanation of confusion matrix, true positives and false negatives should be maximized, whereas true negatives and false positives should be minimized, so that sensitivity of adopted algorithms will be effective on the tested features. Accuracy is not enough to describe performance of model-1; however, we evaluated model-1 according to other metrics. We address this problem using the evaluation metrics in Equations (33), (34), (37) and (38), respectively. Figure 10 displays confusion matrix of model-1, which illustrated that true positives and false negatives were the largest entries in the matrix, whereas true negatives and false positives were the lowest entries, respectively. Nevertheless, to conform that the results are statistically significant, the *JI* is computed using Equation (38) by counting the number of accuracy of similar classes ≥ 54.476%. Simulation results demonstrated that *JI* is up to 0.5. Thus, the similarity index was rejected, leading to the conclusion that the adopted system was statistically significant. Moreover, in order to assess the imbalanced samples (overoptimistic estimation of the classifier ability on the majority class to be dominant) of the adopted multi-stacked deep BiLSTM network, we evaluated MC index from Equation (34). MC generated a high score only if the multi-stacked BiLSTM recognizer was able to correctly predict the majority of positive feature instances and the majority of negative feature instances. MC ranges in the interval with extreme values {–1 and +1} were obtained in case of perfect misclassification and classification, respectively. The MC in this case achieved an average score of 0.949. MC computed results show that the adopted network was able to successfully classify the new input features without minority or majority class bias, reporting only four false negatives (But, Angry, Car and Please), whereas four ASL words (Again, Clothes, Excuse and Go) in the feature vector were all correctly classified (for $\varphi_{1}$ = 0 or $\varphi_{2}$ = 0), in this case, MC = 1. In FI computation, if each class in training feature perfectly matches with class in testing feature, then FI is 1, while if each class in training feature is equally shared over the entire classes in testing feature, then FI is 0. Therefore, FI index achieved good matrix overlap of 0.723 in Table 8. Furthermore, model-1 was evaluated using BI Formula (37), where the average gauge of the likelihood of the informed decision reveald a score of 0.943. The obtained results are acceptable.

**Figure 13:**
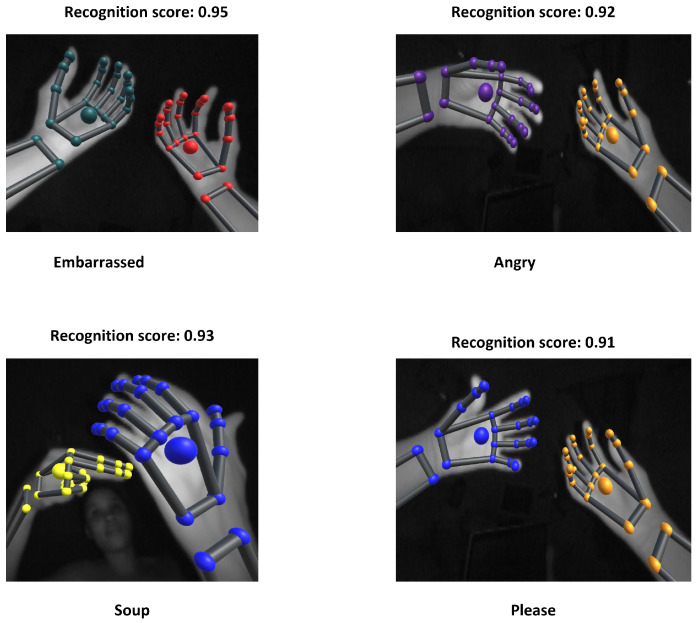
Misclassified words.

Furthermore, in Figure 13 we displayed words with least accuracy: Please, Angry, Friendly, Embarrassed and Soup. ASL word Angry was performed by clawing double hands and inserting fingertips against stomach. Then, hands were forcefully pulled up and outward. ASL word Please or Pleasure was performed by placing both hands on chest, with both palms facing outwards. Then, hands moved in circular motion. ASL word Friendly was performed by raising double hands a few inches in front of head. Then, fingers were wiggled using double hands backward movement. The low accuracy was due to word Please being misclassified as Angry and vice versa. Recognition of these words is thorny, because they share similar considerable parameters.

**Figure 14:**
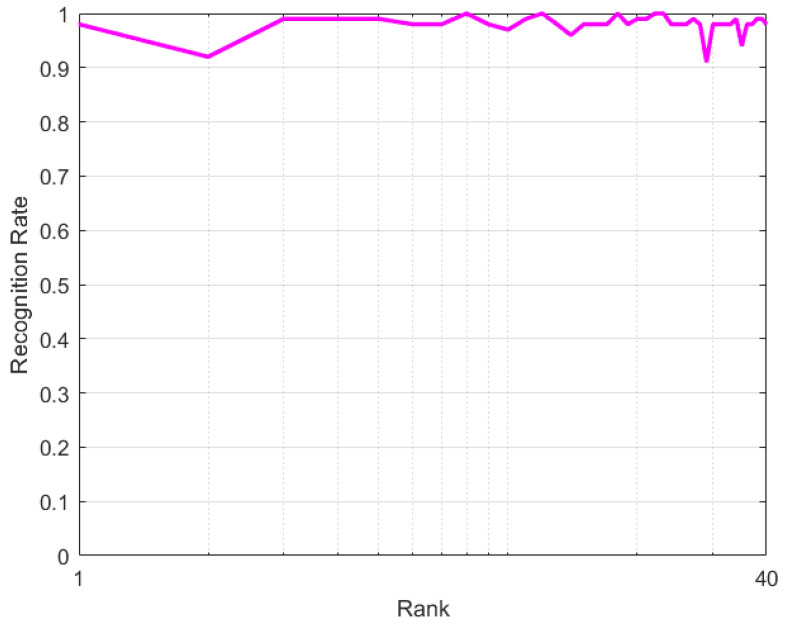
CMC evaluation.

In addition, CMC curve is designed to illustrate recognition rate versus rank score. In this plot, each learning set exempted the knowledge of hand dynamics to measure the similarity contribution of each word combinations. Thus, best recognition was achieved at lower rank, whereas low recognition was achieved from the high rank, as shown in Figure 14. The double-hand ASL words with least ranks were Car (10th), Come (35th), Finish (14th), Go (29th) and Good (2nd). These gestures can achieve best recognition without knowledge of hand dynamics, whereas ranks 8th, 12th, 18th, 23rd and the remaining ranks are difficult to recognize without knowledge of hand dynamics. This demonstrates that not all gestures are unique; each gesture needs different number of discriminating features during recognition. It is worth noting that manual hand features are promising to address misclassification. However, it is difficult to design network suitable for all the dynamic hand gestures. To overcome this challenge, there is need to design network that has a series of concatenated classifiers, so that each group of gestures could have a suitable classifier, as well as features.

## 5. Conclusions

In this work, we addressed the misclassification problem of double-hand dynamic similar and non-similar ASL words. The method achieved an average a recognition accuracy of 97.983% when aiding an effective and automatic recognition of complex double-hand dynamic ASL words from 3D skeletal hand-joint video features of hand motion trajectories and pause, which were developed inside a multi-stacked deep BiLSTM enhanced with machine learning tools. The proposed method designed a consolidated input feature vector. Our method outperformed the existing state-of-the-art methods. Although we experienced misclassification of a few words, it is worth emphasizing that multi-stacked deep BiLSTM initialized from transfer learning with multi-features is promising with regard to challenging, small and large vocabularies of static and dynamic sign words. In a nutshell, misclassification of double-hand dynamic gestures and general gestures could be addressed by extending the vocabulary to accommodate more gestures with various complexities. In addition, if we are to consider the real application of sign-language recognition, then the recognition network should be trained on a relatively small number of gestures, and recognition could be treated as a multi-feature problem. This work can be applied to ubiquitous SLR systems, mobile games, and robotics. Further research should investigate spatial information from skeletal hand-joint video frames to address the misclassification of dynamic sign words.

## Figures and Tables

**Table 2:** Results of correlational analysis.

	Shape	Motion	Position	Angles	Pause	Relative Trajectories
Shape	1					
Motion	0.9444	1				
Position	0.8781	0.9351	1			
Angles	0.86728	0.93985	0.84453	1		
Pause	0.87361	0.71931	0.90719	0.89857	1	
Relative trajectories	0.95351	0.94203	0.89075	0.90681	0.81375	1

**Table 3:** Extracted features.

Features	Point of Interest	Description
Angle points	Pitch, yaw and roll	Hand orientation; 44 skeletal hand joints
Relative trajectories	Motion	Hand motion trajectories, frame speed and velocity.
Positions	Direction	Arm, palm, wrist and five fingers; (thumb, index, middle, ringy and pinky)
Finger joints	Coordinates	Coordinate of five fingers’ tip, metacarpal, proximal, distal and interdistal.
Hand pause	Motion	Hand preparation and retraction.

**Table 4:** Network parameter selection.

Network Layers	Parameter Options	Selection
Input layer	Sequence length	Longest
	Batch size	27
	Input per sequence	170
	Feature vector	1 dimension
Hidden layer	3 Bi-LSTM layers	Longest
	Hidden state	200
	Activation function	Softmax
Output layer	LSTM model	Many to one
	Number of classes	40

**Table 5:** Execution environment.

Deployment	Descriptions
PC	Dell
	CPU: Intel Core i7-9th Gen
	Memory Size: 8 GB DDR4
	SSD: 500 GB
LMC sensor	Frame rate: 64 fps
	Weight: 32 g
	Infrared camera: 2 × 640 × 240
	Range: 80 cm
	$150\times 120^{\circ }$
Participants	Ten
Selections	40 ASL words
	10 times number of occurrence

**Table 6:** Dataset description.

Classes	Amount
Frames	524,000
Samples	4000
Duration (sec)	8200

**Table 7:** Proposed models combination.

Models		Epochs	Execution Time (s)
Features Combination	Model Depth		
Shape + Motion + Position + Angles +	3-BiLSTM layers	300	1.05
Pause + Relative trajectory			
Shape + Motion + Position + Angles	3-BiLSTM layers	300	1.01

**Table 8:** Performance evaluation of multi-stack deep BiLSTM network using leave-one-subject-out cross-validation.

Word Classes	*Accuracy*	*FI*	*MC*	$S_{v}$	$S_{f}$	*BI*	*JI*
Again	0.98	0.7	1	0.98	0.992361	0.972361	0.494949
Angry	0.92	0.707721	0.842701	0.888889	0.956522	0.845411	0.510638
Available	0.986486	0.582301	0.970193	0.970874	0.994819	0.965692	0.341297
Bad	0.99	0.703562	0.959462	0.99	0.985622	0.975622	0.497487
Bicycle	0.993197	0.579324	0.984886	0.98	0.972569	0.952569	0.335616
Big	0.984772	0.707251	0.969581	0.99	0.979381	0.969381	0.505102
But	0.980198	0.989899	0.489899	0.989899	0.5	0.489899	0.98
Car	1	1	0.899994	1	0.989457	0.989457	1
Cheap	0.975	0.701793	0.943489	0.970297	0.999673	0.96997	0.497462
Clothes	0.97	0.703599	1	0.960784	0.986117	0.946902	0.5
Cold	0.994898	0.716115	0.989841	0.990099	1	0.990099	0.512821
Come	1	1	0.959284	1	0.98884	0.98884	1
Dance	0.975	0.701793	0.963497	0.970297	0.989916	0.960213	0.497462
Embarrassed	0.955	0.709103	0.976592	0.933333	0.979465	0.912798	0.507772
Empty	0.98	0.7	0.965484	0.98	0.969476	0.949476	0.494949
Excuse	0.98	0.7	1	0.98	0.981238	0.961238	0.494949
Expensive	0.98	0.7	0.974596	0.98	0.961296	0.941296	0.494949
Finish	1	1	0.958249	1	0.952948	0.952948	1
Fork	0.975	0.701793	0.976222	0.970297	0.983176	0.953473	0.497462
Friendly	0.993103	0.571305	0.984467	0.979167	1	0.979167	0.326389
Funeral	0.985	0.698221	0.965785	0.989899	0.976355	0.966254	0.492462
Go	1	1	1	1	0.954389	0.954389	1
Good	1	1	0.969829	1	0.965481	0.965481	1
Happy	0.975	0.701793	0.949985	0.970297	0.968367	0.938664	0.497462
Help	0.98	0.7	0.947892	0.98	0.983923	0.963923	0.494949
Jump	0.975	0.701793	0.928965	0.970297	0.976583	0.94688	0.497462
March	0.994898	0.716115	0.989841	0.990099	1	0.990099	0.512821
Money	0.975	0.701793	0.939785	0.970297	0.954873	0.92517	0.497462
Please	0.912458	0.485965	0.801818	0.930233	0.905213	0.835446	0.274914
Pray	0.984694	0.705419	0.969432	0.989899	0.979381	0.96928	0.502564
Read	0.98	0.7	0.985672	0.98	0.972345	0.952345	0.494949
Request	0.98	0.7	0.925498	0.98	0.991438	0.971438	0.494949
Sad	0.979899	0.712525	0.960582	0.961165	1	0.961165	0.507692
Small	0.989712	0.444416	0.968427	0.95	1	0.95	0.197505
Soup	0.935	0.694709	0.977349	0.92233	0.965481	0.887811	0.494792
Spoon	0.979452	0.573573	0.954375	0.97	0.984375	0.954375	0.33564
Time	0.98	0.7	0.982396	0.98	0.974928	0.954928	0.494949
Want	0.994819	0.714435	0.989686	0.989899	1	0.989899	0.510417
What	0.994819	0.714435	0.989686	0.989899	1	0.989899	0.510417
With	0.984694	0.697926	0.969432	0.979381	0.989899	0.96928	0.489691
AVERAGE	0.979827	0.723467	0.949372	0.974941	0.967648	0.942588	0.54476

**Table 9:** Performance accuracy of the developed models.

Network Models	Top-1	Top-2	Top-3	Features Combination	Model Depth
Deep BiLSTM	0.954	0.971	0.989	Shape + Motion + Position + Angles + Pause + Relative trajectory	3-BiLSTM layers
Deep BiLSTM	0.912	0.929	0.945	Shape + Motion + Position + Angles	3-BiLSTM layers

**Table 10:** Comparison of adopted Deep Bi-LSTM with a state-of-the-art method.

Methods	Type of Deep Learning	No. of Epochs	Depth of LSTM	Convergence Rate	Execution Time
Avola et al. [32]	Deep Bi-LSTM	800	4 units	100,000 iter	not reported
Our proposal	Deep Bi-LSTM	300	3 units	10,000 iter	GPU 1002

**Table 11:** Performance validation of Multi-stack deep BiLSTM from adopted optimization scheme.

Optimization Scheme	Accuracy (%)	Precision (%)	Recall (%)	F1-Score (%)
AdaGrad	94.701	94.006	94.869	94.003
SGD	95.011	94.998	95.01	94.786
Adam	97.983	96.765	97.494	96.968

**Table 12:** Performance comparison of the multi-stacked BiLSTM network with method in [32].

	ASL Data Set			
Approach	Accuracy (%)	Precision (%)	Recall (%)	F1-Score (%)
Avola et al. [32]	96.4102	96.6434	96.4102	96.3717
Ours	97.881	98.007	97.879	97.998
	**LMDHG Data Set**			
Avola et al. [32]	97.62			
Ours	97.99			
	**SHREC Data Set**			
	Accuracy (%)			
	14 Hand Gestures	28 Hand Gestures		
Avola et al. [32]	97.62	91.43		
Ours	96.99	92.99		

**Table 13:** Performance of the multi-stacked BiLSTM network initialized with data-driven optimization applied to SHREC data set.

Approach	Algorithms	Accuracy (%)	
		14 Hand Gestures	28 Hand Gestures
De Smedt et al. [65]	SVM	88.62	81.9
Boulahia et al [69]	SVM	90.5	80.5
Ohn-Bar and Trivedi [70]	SVM	83.85	76.53
HON4D [71]	SVM	78.53	74.03
Devanne et al. [72]	KNN	79.61	62
Hou et al. [73]	Attention-Res-CNN	93.6	90.7
MFA-Net [74]	MFA-Net, LSTM	91.31	86.55
Caputo et al. [75]	NN	89.52	-
DeepGRU [76]	DeepGRU	94.5	91.4
Liu et al. [68]	CNN	94.88	92.26
Li et al. [67]	2D-CNN	96.31	93.33
Ours	Multi-stack deep BiLSTM	96.99	92.99

**Table 14:** Performance of the multi-stacked BiLSTM network initialized with data-driven optimization applied to LMDHG data set.

Approach	Algorithms	Accuracy (%)
Boulahia et al. [64]	SVM	84.78
Devanne et al. [72]	KNN	79.61
Lupinetti et al. [35]	CNN-ResNet50	92
Hisham and Hamouda [26]	Ada-boosting	91.2
Ours	Multi-stack deep BiLSTM	97.99

## Data Availability

American sign language data set of dynamic words accessed (10 May 2021) at https://bitbucket.org/visionlab-sapienza/2018-jrl-ieee-tmm_-application_dataset/sr. LMDHG data set is accessed (13 May 2021) at https://www-intuidoc.irisa.fr/en/english-leap-motion-dynamic-hand-gesture-lmdhg-database/. SHREC’17 data set is accessed (13 May 2021) at http://www-rech.telecom-lille.fr/shrec2017-hand/.
